# The ‘Is’ and the ‘Ought’ of the Animal Organism: Hegel’s Account of Biological Normativity

**DOI:** 10.1007/s40656-022-00498-8

**Published:** 2022-04-29

**Authors:** Luca Corti

**Affiliations:** University of Padua, Piazza Capitaniato 3, 35139 Padova, Italy

## Abstract

This paper investigates Hegel’s account of the animal organism as it is presented in the *Philosophy of Nature*, with a special focus on its normative implications. I argue that the notion of “organisation” is fundamental to Hegel’s theory of animal normativity. The paper starts by showing how a Hegelian approach takes up the scientific image of organism and assigns a basic explanatory role to the notion of “organisation” in its understanding living beings. Moving from this premise, the paper turns to the group of accounts in contemporary theoretical biology known as “organisational accounts” (OA), which offer a widely debated strategy for naturalizing teleology and normativity in organisms. As recent scholarship recognizes, these accounts explicitly rely on insights from Kant and Post-Kantianism. I make the historical and conceptual argument that Hegel’s view of the organism shares several basic commitments with OAs, especially regarding the notion of “organisational closure”. I assess the account of normativity that such accounts advance and its implications for how we approach Hegel. Finally, I argue that the notion of “organisation” is more fundamental to Hegel’s theory of animal normativity than the Aristotelian notion of “Gattung” or “species”, which by contrast appears derivative – at least in the *Philosophy of Nature* and the *Lectures* – and does not play the central role in his account maintained by some scholars.

## Introduction

This paper investigates Hegel’s account of the animal organism as it is presented in his *Philosophy of Nature* and *Lectures*, focusing in particular on its normative implications. The question of the constitution of the living organism and its normativity is key to understanding the Hegelian answer to the broader question of the relationship between *norms* and *nature*, an issue that has been central to a number of debates (which extend well beyond Hegel) and enjoyed such import that it now hardly needs to be introduced^1^.

The contemporary relevance of Hegel’s views on norms has emerged thanks to many readings of Hegel’s philosophy (and resulting Hegelian theories of agency, conceptual content, judgment, etc.) guided by an understanding of his notion of *Geist* as a distinctly normative realm. Recently, however, scholars have increasingly pointed to the fact that “normativity” for Hegel is not something exclusively restricted to the social or spiritual domain. Rather, Hegel identifies various forms of normativity *in* nature, which are exhibited paradigmatically by the living organism. To understand how norms can be considered a constitutive part of the natural domain thus requires special focus on the animal organism. In this paper, I explore Hegel’s contention that “organisation” is a basic feature of living beings. Reconstructing and illuminating this key insight will allow me to show how and why “organisation” is the central notion shaping Hegel’s views on organism and the natural normativity they manifest.

I will moreover argue that Hegel’s views on ‘organisation’ are not merely an issue of historical interest. To foreground their potential for the current debate around norms, I will put them in conversation with a group of accounts in contemporary theoretical biology referred to as the “organisational account” (OA) or “biological autonomy” view and the philosophical discussion surrounding them. This group of theories offers a particular take on biological phenomena, especially organisms, and in an attempt to naturalize functions advocates a definition of *natural normativity* that has been widely debated (Moreno & Mossio 2015; Mossio et al. 2009; Artiga 2011; Artiga & Martínez 2016; Garson 2017; Montévil & Mossio 2015; Bich & Mossio 2011; Mossio & Bich 2017; Mossio et al. 2016). At the same time, there have been several recent attempts to locate the genealogical origins of the OA biological tradition by positioning Kant as its founding father and a key proponent of its core concepts (Mossio & Bich 2017; Cooper 2018; Hunemann 2017; Kreines 2005, 2015; Ginsborg 2006, 2014; McLaughlin 2001; Weber & Varela 2002). As my analysis will show, we can also read Hegel as advocating an organisational approach and thus as belonging to this same tradition. I will elaborate how the OA accounts enable a deeper understanding of some interesting aspects of Hegel’s thought, especially its position on normativity in animal organisms^2^.

Starting from this premise, my paper will proceed in three steps:

1. I will begin quite far from Hegel by outlining the OA conceptual framework and its status within the field of current theoretical approaches to the living organism.
2. I will show how some key OA notions can be historically and conceptually connected to Hegel’s position and used to shed light on his views about biological phenomena, including their normative dimensions.
3. I will outline and discuss the OA’s strategy for naturalizing normativity and its main problems, which have emerged in recent scholarship. I will present the Hegelian response to these issues, which will enable me to specify his views and further define his account of natural norms^3^.

## Organism and organisation in biology

In recent decades, various dissatisfactions with neo-Darwinian and standard evolutionary theory have led biologists and philosophers of biology to give renewed attention to the notion of “organism”, which has reentered the lexicon of biological theory as an important unit of analysis. Traditionally considered a derivative category subordinate to the notion of adaptation, “organism” has become a central player in biological theories (Gilbert & Sarkar 2000; Bich & Damiano 2008; Walsh 2015, Huneman 2010; Nicholson 2013, 2014; Toepfer 2012; Toepfer & Michelini 2016; Cornish-Bowden & Cárdenas 2019), with theoretical biologists and philosophers advocating for its theoretical relevance and defending the need to put it back on the agenda (Pepper & Herron 2008). Though it played virtually no role in evolutionary discussions, especially in the formation of the Modern Synthesis (Walsh 2015, 217), new interest is now being focused on “organisms” instead of primarily on supra-organismal units (populations) or sub-organismal entities (genes).

As these insights have gained traction, a set of theoretical models has emerged that makes the claim that organisms cannot be accounted for merely by looking at their parts; instead, we should think of them as particular kinds of *systems*. This shift in perspective has come to be identified with a distinct line of inquiry in biology. According to its proponents,

unlike the evolutionary approach, the organisational one puts more emphasis on the internal dimension of living systems rather than on external influences. (Mossio & Bich 2017, 10)

In this theoretical context, organisms are often understood as particular kinds of *adaptive* systems understood in terms of “biological autonomy”, “biological self-organisation,” and “biological self-determination.”

Autonomy is a property of widespread biological significance; living organisms in general are autonomous systems, as are reproductive lineages, species, and some kinds of biological communities. (Christensen & Bickhard 2002, 4)

Philosophical discussions around this framework pose questions regarding the ontological definition of the organism, epistemological questions regarding the conditions for its identification, and conceptual questions regarding its defining properties (Meincke 2019, Bich 2012, Christensen & Bickhard 2002, Walsh 2012, 2015).

In discussing this family of views – about whose origins I will speak later – I will focus specifically on one well-remarked upon member: the *organisational account* (OA), which represents the most recently developed and fully elaborated view^4^. In fact, the OA not only provides an account of the “organism” but also attempts to naturalize teleology, normativity, and functionality. In the words of its proponents, “in the autonomous perspective an organisation is by definition closed and functional” (Moreno & Mossio 2015, 72). Starting from this insight, the OA claims it can “adequately naturalize teleology and normativity” (Mossio et al. 2009, 816). Proponents have stressed the relevance of Kantian insights, especially the notions of *Selbstzweck* and organisation, to this approach and have presented the OA as offering a well-defined and viable definition of the animal organism. Against this background, it becomes clearer why the conceptual issues surrounding the OA are not only interesting in themselves but might also be productive for approaching Hegel’s theory of organism and his notion of natural normativity at the level of animal life.

### The organisational account: the basics

The core idea underlying organisational accounts is that organisms are a particular *kind* of self-maintaining system. What distinguishes them from other self-maintaining systems present in nature is that their basic properties are “inherently related to self-determination.” (Moreno & Mossio 2015, 1) Self-determination is thus crucial, since it “remains the conceptual core of autonomy” (Ibid., xxix). What does the notion of autonomy mean here? How is it spelled out in naturalistic terms?

Proponents of the OA maintain that biological self-determination is characterized by two fundamental properties, which they term “organisational differentiation” and “organisational closure”.

i) “Organisational differentiation” is a property of systems that are constituted by topologically different localizable structures or components, some of which are “generated” by the system itself (I will discuss this in a moment) (Mossio et al. 826).

ii) “Organisational closure”, on the other hand, specifies the particular relation of reciprocal dependence among parts of an organisationally differentiated system. To introduce this notion, the OA makes a further conceptual distinction between “process” and “constraints” (Moreno & Mossio 2015, ch. 1; Montévil & Mossio 2015; Bich & Mossio 2011; Mossio & Saborido 2016.)^5^

The core insight behind this second distinction is relatively simple: some parts of an organized system play the role of “constraints” when they act upon a given process (i.e., exert a causal role on it) and maintain a certain degree of independence with respect to the process itself during the relevant time scale (during which the process occurs). Constraints are elements that enable the occurrence of a process but are “not altered by (i.e. [are] conserved through) that process at the scale at which the latter takes place” (Montévil & Mossio 2015, 182)^6^.

The paradigmatic examples of the notion of “constraint” – which are fundamental to the development of the whole model – are metabolic processes, i.e., processes in which enzymes prompt a catalytic reaction. Considered at the right level of description, enzymes can be seen to play a causal role in the process of a particular chemical reaction (i.e., enable the reaction to occur) without being “consumed” or “altered” by the reaction itself.

Metabolic reactions exhibit an additional feature that makes them paradigmatic: functioning as constraints vis-à-vis processes, enzymes are also generated by the organism via processes that are in turn “constrained” by some other element that is not altered in the process.

They act on processes (enzymes catalyse reactions) and, at the same time, they are produced by other efficient causes (enzymes are produced by other metabolic processes within the cell) (Mossio & Bich 2017, 14 fn 15)^7^.

Enzymes are foundational for the organisational account because they exemplify a basic feature of “constraints”: they “depend on” each other and contribute to the maintenance of a system.

The relation among constraints is spelled out in the OA in various ways: sometimes in terms of “dependence”, sometimes in terms of “conditions of existence” (Bich 2016, 204 ff., Saborido et al. 2011, 584) or reciprocal “presupposition[s]” (Saborido & Moreno 2015). It is from this idea of a mutual “dependence of constraints” or “presupposition” among them that the core notion of OA theoretical accounts emerges, namely the idea of “organisational closure”^8^. Closure is a property of a system in which.

the existence of each constraint depends on the existence of the others, as well as on the action that they exert on the dynamics. In this kind of situation, the set of constraints realizes self-determination as organisational closure. (Mossio & Montévil 2015, 181)

When constraints collectively contribute to the maintenance of the system, and each constraint depends on at least one other constraint, there is closure. In this regard, metabolic reactions are again paradigmatic:

Metabolic organisation consists of a network of reactions, finely regulated by their highly complex material components (enzymes), and regenerated by the very network that they control in an organisationally closed way. (Mossio et al. 2009, 827)

The proponents of this view propose “closure” as the benchmark or criterion for differentiating biological systems from their environments (Mossio & Montévil 2015, 187)^9^.

A third property central to the OA conceptual framework – which I will not consider in detail here but which is potentially interesting for approaching Hegel – is.

(iii) “interactive openness”. For the proponents of OA, “autonomy should not be confused with independence: an autonomous system must interact with its environment in order to maintain its organisation” (Moreno & Mossio 2015, xxviii). This claim is premised on the idea that such systems need energy to maintain themselves, i.e., they are far-from-equilibrium not equilibrated thermodynamic systems.

The conceptual distinction between “constraints” and “processes” therefore enables the OA view to distinguish between *closure* and *interaction* in the following terms:

While biological systems are (by hypothesis) closed at the level of constraints, they are undoubtedly open at the level of the processes, which occur in the thermodynamic flow. Autonomous systems are then, in this view, *organisationally closed* and *thermodynamically open.* (Moreno & Mossio 2015, 6)

According to its proponents, this model can be of explanatory use for a wide range of phenomena, because it is relatively simple while at the same time providing a blueprint for approaching various forms of biological complexity.^10^

What has most interested philosophers about this model is how advocates have seen it as yielding a new account of the normativity of functions. Proponents of the OA have defended the idea that function attribution and its normative import in biological systems are best accounted for in terms of the roles different elements play within regimes of organisational closure (Mossio et al. 2009; Moreno & Mossio 2015; Mossio & Bich 2017).

Closure is then what grounds functionality within biological systems: constraints do not exert functions when taken in isolation, but only insofar as they are subject to a closed organisation. (Mossio & Montévil 2015, 186)

In their words,

For each given class of self-maintaining systems, the *primary* function Fp of T is the contribution of T to the self-maintenance of S that is subject to closure in the more basic regime of self-maintenance. (Mossio et al. 2009)

The OA account has emerged as one of the leading groups of approaches to describing functions and their normativity (cf. Garson 2016). It identifies the function of a trait in terms of the role that trait plays in a self-maintaining, closed system. In short: “constraints subject to closure correspond to biological functions” (Mossio & Bich 2017, 16). The normativity implied by functional attribution (what an item “ought” do) thus derives from the basic intuition that, without the feature’s performance of its attributed function, the organisationally closed self-maintaining system would collapse. According to this view,

Closure is the circular causal regime that adequately grounds intrinsic teleology and, consequently, normativity. (Mossio & Bich 2017, 16)

In this way, the OA attempts to naturalize functionality and normativity in a well-defined and scientifically viable account of biological systems whose constitutive feature is a self-maintenance that underwrites function ascriptions as well as “ought”-ascription. According to proponents, the ought-ascriptions thus performed are ‘objective’, since they represent a form of “non-observer-dependent normativity” (Saborido & Moreno 2015).

## Hegel and the organisational account of the organism

In recent years, proponents of the OA have looked to history in various ways in attempt to locate the origins of their views. This has led to the identification of a series of episodes in what advocates call the “prestigious history in philosophy of science and theoretical biology” (Mossio & Bich 2017, 12) in which organisation was examined in attempt to naturalize normativity. The figure who appears most frequently in these genealogical reconstructions is Kant, who is often given the role of founding father.^11^ In fact, Kant’s notion of *Selbst-zweck* and related views on self-organisation have attracted much attention from both philosophers of biology and historians (Ginsborg 2006; Kreines 2005, McLaughlin 2014, Kauffman 1993) 12. I will not dwell on this topic, which has already been covered thoroughly in the literature, but rather will build upon this existing scholarship to investigate how Hegel similarly takes up and develops some key OA insights. I will thus focus on the following question: can the OA help illuminate some of Hegel’s views? If so, what kind of OA position might we attribute to Hegel?

My claim will be that several of the OA views presented above can help us interpret issues at stake in Hegel’s *Philosophy of Nature*, bringing to light some crucial features that Hegel attributes to organisms. In particular, the OA enables us to see Hegel as isolating a level of normativity that precedes his discussion of *Gattung* (i.e., genus or species). To clarify this point, I will sketch out some of the key features of Hegel’s account of organisms and show how they can be illuminated by OA insights:

(i) First, it is worth noting that the notion of “process” plays a crucial role in Hegel’s understanding of biological phenomena. In fact, Hegel defines living beings as basically processual in nature. He claims that organisms are “essentially process” (GW, 24,2 925) and “the organism is … the infinite self-stimulating and self-sustaining process” (PN § 336). Hegel thus appears to embrace a basic *processual ontology of the organism*^13^.
(ii) Yet he notoriously also adds that this process should be understood as a form of self-differentiation, i.e., as a process in which the organism organizes itself into various parts. Although this process only becomes explicit and fully developed in what Hegel considers the highest organismal form, the *animal organism*, he identifies differentiation as a mark of life and property that can be found in all forms of living organisation, including ‘lower’ forms such as “plants” (which organize themselves “into mutually distinct parts”, PN § 343.)^14^
(iii) For Hegel, self-differentiation in living beings must ultimately be understood as ontologically derivative and dependent on process. Referring to the material parts of a system, he notes that “their existence is the process in itself” (PN § 342 A); they “do not exist outside of it” (GW 24,2, 1141; GW 24,1, 132). Hegel moreover stresses that such parts change and that some can appear and vanish in different time scales: “The members are destroyed as well as engendered” (PN, § 341 A).
(iv) Notably, for Hegel (as for Kant) self-differentiation in the living organism is not reducible to *mere* material complexity. Rather, it corresponds to a form of what the OA calls “organisational differentiation”. These differentiated parts are characterized primarily in terms of their *roles* in the process, not by their material compositions or topologies.

These four features help define Hegel’s conception of the notion of “organisation” (*Organisation*) and provide a clearer understanding of his claim that organisation is the hallmark of the living being^15^.

But how does Hegel specify the roles involved in such “animal organisation” (GW 24,1 176)?

Here the OA (together with Kantian insights) might be of use, directing our attention to some key elements of Hegel’s account. The first is that when Hegel examines the *internal* differentiation of an organism, he distinguishes between two kinds of items: “the internal has means and has material … these means are … the organs, the members” (GW 24,2, 948).^16^ His description refers to parts of systems that act upon certain “material” and identifies the products of such activity as in turn involved in other processes. In his 1828 *Lectures on the Philosophy of Nature*, Hegel exemplifies the point in this way:

Every organ secretes and what is secreted is taken up from other organs, the other organs nourish themselves from the secretions, every organ is *Zweck* und *Mittel*… so is the life of an organ in itself this activity. … Through this process every organ is maintained as a member of the whole. (GW, 24,2, 1153)

I do not think it would be against the spirit (or perhaps even the letter) of Hegel’s text to gloss this distinction as recognizing some functionally organized *processes* (which Hegel calls *Material*) acted upon by an element that plays a role analogous to that of a *constraint* (what he calls *Mittel*).

Before addressing the question of the interdependence of such *Mittels*, and therefore diving into the finely grained structure of Hegel’s account of self-maintenance, let me briefly recall the place of *Mittels* within Hegel’s description of the organism as it is presented in his *Philosophy of Nature*. Hegel’s general account of the animal organism distinguishes between three of its dimensions: (i) its “shape process” or “process of formation”, which corresponds to the internal or physiological structure of the organism; (ii) its “process of assimilation”, which accounts for the organism’s relation with its environment, or what we have called its “interactive openness”; and (iii) its “genus process” (*Gattungsprocess*), which involves reproduction in terms of the preservation of a particular species. Hegel’s discussion of organisationally closed inner structures and components of living systems mainly takes place in relation to the first dimension, “shape” (*Gestalt* PN § 346). For Hegel, parts are organized in three anatomically distinct but integrated sub-systems, each of which has material components – which Hegel calls the “nervous system”, the “circulatory system,” and the “digestive system” (PN § 354).

The key to understanding the functional nature of the components of these systems is their particular kind of dependence on each other. Here Hegel relies heavily on Kant’s insights. In Hegel’s formulation, the logical relation among parts is described using the vocabulary of purpose: “all members are reciprocally momentary *means* as much as momentary *purposes*” (EL §216)^17^. Hegel’s examples in the *Philosophy of Nature* help to illuminate this view and show that something like a relation of causal dependence is central to what Hegel calls the “internal activity (*inner Tätigkeit*)” of a living system (GW 24,2, 948), in the sense that the effects of some organs enable the occurrence of some processes upon which other organs (*Mittels*) can then act. Organisms thus appear to exhibit a property similar to what I described above as “closure of constraints”.

In his *1821/22 Lectures*, Hegel writes.

Every member, every part of organism maintains itself, and it does so at the expense of the others, so that it takes from other parts of the organism, what it needs for itself. (GW 24,1: 454)

In a manner analogous to the OA, the *functional nature* of the items involved is defined not only in terms of their mutual dependence but also in terms of their contribution to the self-maintenance of the organism as a whole. Hegel emphasizes that the various systems (and their parts) constituting the organism are defined in terms of their capacities to jointly contribute to the *general* self-maintenance of the organism. He expresses this point by stating that the systems of sensibility and irritability (together with their material components and structures) are subordinate to those of reproduction – with “reproduction” in this context meaning “regeneration” or self-maintenance (*Selbsterhaltung*) (GW 24, 2 926; WDL 12.186)^18^.

A particular causal regime is therefore distinctive of organisms, whose parts depend on each other in a way that constrains various processes or materials in order to enable self-maintenance. That is what Hegel means when he says

Organic being is actual being which is self-maintaining, and which runs through the process in its own self. These parts bring forth the whole. (PN § 341)

Looking at the detailed structure of self-maintenance in this way allows us to spell out what Hegel means by his references to organisms “creating themselves” or “producing themselves” in a way that appears less problematic than a *causa sui* model of self-creation – or a vital force model (GW 24,2, 908, 949). Interestingly, it appears that Hegel does not understand organisations simply in terms of *operational closure* (Bich 2016) – i.e., as a circular interaction or coupling among organs that each perform their own activities – but rather as a form of “mutual generative dependence between components” (ibid.), to borrow a current phrasing from Bich. Indeed, Hegel seems to frame the activity of some components as a condition for the generation of others. This use of the language of “conditions” is borrowed directly from the scientific discussions of his time and points toward a deeper interdependence among constraints^19^.

What is interesting is that if we can identify such a paradigm in Hegel, then the Hegelian idea of “functionality” and “normativity” that comes into view resonates with the spirit of the OA. As we have seen, in the OA functional attribution can be performed on parts that are members of an organized closed system, i.e., are dependent on other constraints in a closed network. Since their performance is *essential* to the self-maintenance of the system, we can recognize this performance as the *proper* function of the element – or what the item “ought to do”.

The idea of *conditions for self-maintenance* seems to play an important role in the OA account, insofar as it grounds both functional attribution and its normative import: without a given performance, the system would collapse, so performance is what the element (or constraint) *ought to* do. In other words, this performance constitutes a proper function. Proponents of the OA claim that this view “generates a criterion for determining what norms the system is supposed to follow: the system must behave in a specific way, otherwise it would cease to exist” (Mossio & Bich 2017, 18; Saborido et al. 2011, 584).^20^ They underscore that this definition is embedded in a natural-scientific account of living beings. The upshot for a theory of normativity is that, already at this level, it is possible to individuate a basic form of functionality and normativity that is taken to be “natural” – and that can be objectively identified without yet taking into account *either* the organism’s interaction with the environment *or* its reproduction as a member of a species.

If Hegel can be read as sharing some key commitments with this theory, then his account of natural normativity in animal organisms might not *prima facie* involve reference to the notion of “species” or “*Gattung*”, which in fact is addressed in a separate stage of his analysis. This type of normativity precedes and is independent from that of the *Gattung*, since it does not depend “on the contribution that that feature makes to the survival and reproduction of the species” (Mills 2020, 456). Rather, it depends on the contribution that a certain feature makes to a closed organisation, i.e., to the self-maintenance of the organism. Indeed, we can see this line of thought traversing, or even predominant in, his *Philosophy of Nature* and series of five corresponding *Lectures*, which suggests a distinct Hegelian account of norms in nature.

This part of Hegel’s account of the organisational level of normativity is not, as some commentators argue, “historical” (Mills 2020). It is also more fundamental than various other forms of normative activity in the organism (Pinkard 2012; Ikäheimo 2021)^21^. That being said, both Hegel’s theory and current OA views raise further questions: how far can one go with this attribution of “normativity” to organized systems based on the notion of organisational closure (and conditions of existence)? What kinds of phenomena can this sort of view capture, and which can it not? In fact, if one looks closer, the view is not entirely unproblematic, and a set of questions arise that need to be addressed (some of which were clearly not on Hegel’s radar but are essential if we want to articulate a satisfactory Hegelian account). While the attempt to solve some of them might produce other interesting insights into Hegel’s philosophy, some issues appear threatening for both Hegel and the OA. I will raise some of them below before coming to my conclusion. I hope this will help clarify and reinforce my claim that Hegel’s focus on organisation and the normativity tied to it is fundamental to his thought and more prominent than his insistence on the notion of “species”.

## Some criticisms of the Organisational Account^22^

### Limited scope for the attribution of functionality and normativity

The first question regards the scope of normative attributions within this framework: to what kinds of things can we attribute functionality and normativity? Defining the normativity and functionality of an item in terms of its contribution to an organisationally closed system – under the condition that without such a contribution the system would cease to exist – seems to problematically restrict the set of things that can have functions and “ought” to work in a certain manner. This, in turn, prevents us from ascribing functionality to other things that we usually think have a function. There are in fact many parts of an organism that do *not* appear to contribute to self-maintenance as necessary conditions but which we nonetheless take to be functional. An animal organism, like the human one, cannot self-maintain without a heart pumping blood (this is the standard example used in the OA), but it can exist without eyes or ears. However, according to the OA, if eyes are not essential to self-maintenance or part of an organisationally closed system, we are not in a position to say that they have a *function* or “ought to” work in a certain way. This seems counterintuitive. As a response to this problem, the OA introduces the notion of “basic regimes” of self-maintenance to refer to the minimal conditions for a system to exist. This enables it to distinguish between “primary” and “secondary” functions: the former are essential constraints, whereas the latter are tied to more complex forms of organisation. This explanation seems unsatisfactory (Garson 2019; Artiga 2011, 21). Moreover, it *prima facie* counters the fact that some parts of the system are maintained even if they do not perform any activity – as critics point out, “my body would keep maintaining my ears even if they stopped performing any activity (for instance, in deaf people)” (Artiga 2011, 15).

Attribution of functionality to such elements thus might require some additional conceptual resources, such as reference to some notion of “species”, in terms that can account not only for the presence of such parts but also for their proper functions (how they *ought* to operate).

Surprisingly, however, Hegel does not appeal to the notion of “species” or to species-specific considerations to account for these aspects. When he discusses the functionality of some part of an organism, he refers to what he calls “The universal type of the animal” (PN, § 370), framing this “type” as defining the normativity and functionality of existing bodily parts: “In many animals, there are rudiments of organs which belong only to the universal type” (PN, §370Z). As he says,

it is in and from this type that the significance of the undeveloped organism may first be ascertained and assessed. (PN, §352Z)^23^

This “universal type”, however, is not defined in terms of some Aristotelian “nature” but rather as a particular form of *organisation* (more about this in a moment).

### Cross-generational traits

A related issue pertains to one specific sub-class of elements whose effects do not seem to play any role in the self-maintenance of individual systems: so-called cross-generational traits. Ruth Millikan’s paradigmatic example of “sperm” is the most cited one in this context. Sperm does not contribute to the *self-*maintenance of the organism it belongs to, but we *do* think it has a function (or we might want to say that it does). Can we ground such an attribution in an organisational perspective? Or should we embrace a so-called “splitting account” (Delancey 2006), in which the notion of function for these cases gets defined in a different way?

The OA rejects this move and responds to the challenge by stating that cross-generational traits can be well accounted for by the existing notion of organisation. OA theorists defend the idea of using “the very same criterion of individuation of functional traits both for intra-generation and cross-generation ones” (Mossio & Saborido, 2016, 272). What changes, they argue, is the *relevant system* under consideration: in the case of cross-generational traits, the system in question is not the individual organism but rather a broader one that includes both the reproduced and the reproducer. In fact, the two individuals (and the relation between them) can be seen as constituting a particular kind of unitary, organisationally closed system involving “a chain of constraint dependences that unfolds in time beyond the boundaries of a single generation” (Mossio & Saborido 2016, 269). According to this view, a succession of organized beings can be described “as a continuous chain of organized systems connected through constraint dependencies” (Mossio & Pontarotti 2019, 12). The OA’s move thus constitutes an extension of the notion of organisation to encompass multiple organized systems unfolding in time, which are seen as forming a higher-order, “cross-generational organisation” (Saborido et al. 2011; Mossio & Pontarotti 2019). If such an organisation is closed, then we have cross-generational closure and therefore functionality. In this way, traits that do not contribute *prima facie* to the intra-generational organisation and self-maintenance of an individual system (such as ears and sperm) can be seen as contributing to the wider, cross-generational organisation a system that includes offspring^24^.

This view needs certainly to be further qualified, as it raises questions regarding how to conceptually distinguish among kinds of organisation (what differentiates an *intra-generational* organized system from a *cross-generational* one?) and how to establish the relevant time scale to identify a cross-generational organisation (how many generations are needed?). Some of these issues have been addressed in recent literature, while others are still open and need to be discussed. For my purposes, what is noteworthy is how OA authors in this context make some claims that might be of interest for the Hegelian perspective. They maintain, for instance, that a cross-generational organisation “continuously sets the conditions enabling its own reestablishment”, adding that “because of the enduring influence on its own conditions of existence, it is legitimate to claim … that a particular biological organisation never ceases to exist” (Ibid, 13). This kind of structure can be of use in interpreting Hegel’s logical notion of temporality involved into cross-generation^25^.

### Malfunction

Finally, we need to consider the notion of malfunction, which is problematic for the OA. By defining function in terms of an *actual* contribution to an individual regime of self-maintainance, the OA has difficulty distinguishing “malfunctioning” from “not having a function at all” (or, put differently, distinguishing *having* a function from *performing* that function, cf. Garson 2017). This is because its framework suggests that if an item stops performing its proper activity, we should, *strictly speaking*, say that it no longer has a function, not that it is malfunctioning. In this context, the definition of an “appropriate” contribution to the system becomes highly problematic, and the need for further theoretical elaboration reappears.

To this objection, OA theorists reply by employing the idea of various configurations or regimes of self-maintenance: they stress that a system is capable of adopting various regimes according to different demands. In each of these regimes, the constraints can work differently (Saborido & Moreno 2015)^26^. This is called *adaptivity* (Ibid. 89). The basic insight here is that the process of adaptation is made possible by the fact that a trait can work with a specific *range* of variations. Ascriptions of normativity depend on this range, such that when a trait works outside of the range – in a way that inhibits or hinders adaptivity – it can be said to be malfunctioning.^27^

This idea of various regimes of self-maintenance is not unproblematic^28^, but it is interesting for understanding Hegel, who also includes a notion of adaptivity in his theory.

## Hegel’s organisational response to these criticisms and resulting view of norms

We might expect many questions along these lines to be off Hegel’s radar, but this would be incorrect. Indeed, asking them illuminates his stance on crucial issues that are essential to articulating a comprehensive Hegelian view on norms and organisation. In this regard, I will stress two aspects of Hegel’s answer to the above-mentioned criticisms before coming to my conclusion. Hegel’s reply in fact places the notion of organisation at its core: (a) first, he states that an organism is constituted by a multiplicity of systems and uses this idea to address the problem of “malfunctioning”; then (b) he refers to the notion of the “Universal Type of the Animal”, which I briefly touched upon above.

In other words, Hegel’s response to the problem of malfunctioning invokes the central role played by his definition of the organism as a “system of systems”: in Hegel’s perspective, it is one of these sub-systems that can be said to be malfunctioning – not their single parts – and this occurs when the sub-system in question operates in a way that fails to contribute to the maintenance of the whole organism (or perhaps even hinders such maintenance). Here is Hegel’s definition:

The organism is in a *diseased* state when one of its systems … establishes itself in isolation, and by persisting in its particular activity in opposition to the activity of the whole, obstructs the fluidity of this activity, as well as the process by which it pervades all the moments of the whole. (PN §371)

The notion of regime, seen above, might be of use to clarify this point. In Hegel’s account, malfunction is understood as a particular configuration not of a single organ but rather of a particular sub-system, such that the sub-system does not contribute to (or even obstructs) the global self-maintenance of the organism. This malfunctioning in turn alters the “succession of functions” (PN, §372Z). This insight *prima facie* maintains the idea of organisational closure as a basic feature of the organism and enables us to distinguish “malfunctioning” (when a member is part of an system that does not contribute to general self-maintenance) from “being not functional” (when the item is not part of an organisationally closed system in the first place). Towards this end, Hegel introduces the idea of a *right kind* of interdependence among sub-systems that is distinguishable from a *wrong kind* that obstructs the general self-maintenance of the organism: “illness is basically the isolation of a system, of a mode of activity” (GW 24,1 467, cf. also GW 24,2 1153). Whatever the advantages of this view in solving the issue of “malfunctioning,” Hegel’s approach to malfunction needs to be differentiated from an account of “defectiveness” in terms of the correspondence of an item to a particular *Gattung* or species (understood in an essentialist fashion). In fact, his definition of “malfunctioning” – particularly in the paragraphs regarding “sickness” (PN372) – does not appeal to the notion of “species” but rather centers on the contribution of a particular system to the general self-maintenance of organisms in terms of functional closure^29^.

As a part of his definition of the right kind of functionality, Hegel introduces the notion of the “Universal type of the animal” (PN §370, §368Z) as the standard against which we can normatively assess not only sickness but also various forms of animality in nature (“from the simplest to the most perfect”). The notion, however, is defined once more in terms of a particular *kind of organization*.

The universal *type of the animal* determined by the Notion, lies at the basis of the *various forms* and *orders of animals.* This type is exhibited by nature partly in the various *stages of its development* from the simplest organisation to the most perfect. (PN §370, in original §368)

Again, the notion of organisation appears to be fundamental – so much so that for Hegel the “universal type of the animal” should guide the inquiry of the natural scientist (Ibid.). Sometimes *Gattung* or species appears to be defined in terms of this notion (PN §368Z).

Notably, Hegel also considers cross-generational relations among individuals in terms of *Gattung*. It remains unclear, however, whether Hegel’s notion of *Gattung* can be fully spelled out in terms of a higher-order cross-generational organisation (i.e., as a continuous organized chain of reproducing and reproducing beings). A fuller analysis of this issue would take us too far from our argument and is beyond the scope of my paper. Speaking generally, however, the relative absence of *Gattung* in Hegel’s discussion of normativity in the *Philosophy of Nature* – and the notably little space Hegel devotes to it in the text and in his Lectures (as well as in the *Logic*) – is quite surprising, especially when compared to his discussion of “organisational” aspects. In some passages, Hegel appears to consider the distinctions among *species* in the animal realm to be primarily an empirical matter. In the *Science of Logic*, he famously writes that it is *not* in the concept of animal in general that.

one can find the determinations according to which animal in general is divided into mammal, bird, etc., and these classes are then divided into further genera. Such determinations are taken from elsewhere, from empirical intuition; they come to those so-called concepts from without. (WDL, 21.45, 38)

Hegel not only maintains that classification of the animal realm into various species will never be “complete” but sometimes – as in this passage – seems to go so far as to suggest that the content of the notion of “species” is largely grounded in contingent empirical facts. What does the idea of *Gattung* add to his account of the animal organism?

One could begin replying to this question by noting, as I did above, the presence of some elements or traits in animal life that cannot be accounted for in terms of organisational closure and instead require a different level of description that involves the relation between a *specimen* and the *species* to which it belongs. I will not dwell on this topic here, since my point, as I hope to have shown, can be made independent of it. Indeed, what we have seen so far seems sufficient to correct the view that the notion of species is *the* central concept in Hegel’s understanding of life and its normativity and to support the alternative view that Hegel attributes “organisation” and the kind of normativity it entails a more basic role in his account.

## Conclusions

I have tried to investigate some parts of Hegel’s *Philosophy of Nature* by putting them in dialogue with current discussions in the philosophy of biology. I do not think that we can say, as Klaus Brinkmann does, that Hegel’s entire account of organism “seems to agree well with what we would expect in a modern biology course book” (Brinkmann 1998, 141), since many of Hegel’s views on animal organism sound implausible and hopelessly outdated. Still, as I hope to have demonstrated, there is a conceptual core animating the Hegelian account of organism that is of interest. In my reading, Hegel’s view assigns a constitutive role to the notion of “organization” and can be identified as belonging to the philosophical tradition that make organisation a crucial property of the living. Hegel’s views are both historically and conceptually tied to some views in current theoretical biology – in particular to the OA, which attempts to conceive of organisms as organisationally closed systems that assimilate external material in order to fuel their metabolic processes (and structures) of self-maintenance.

As I have shown, these contemporary views help illuminate some passages in Hegel, and, more interestingly, account for some *normative properties* that characterize a particular sort of biological organisation. In fact, reference to the OA can direct us to certain crucial points in Hegel and help us see that the emphasis in the *Philosophy of Nature* on organisation as a constitutive mark of life is fundamental to Hegel’s account of biological normativity (including his views on *malfunctioning* and *sickness*). If the OA proves sufficiently explanatory in this regard, then it might represent a strategy for isolating a particular kind of normativity involved in contemporary natural scientific discourse that is Hegelian in spirit^30^. Such an account might weaken Sellars’ “naturalistic ‘thesis’ that the world can, ‘in principle,’ be described without using the term ‘ought’ or any other prescriptive expression” (Sellars 1957, § 79) and help move us towards a perspective in which a particular kind of “normativity” is involved in at least some sorts of biological complexity (as demonstrated by explanatory frameworks like the OA). On the other hand, looking at Hegel can enable us to tackle and improve some of the disputes surrounding the organizational accounts by raising some interesting challenges to these views (for instance, by pointing to the relevance of the idea of *Gattung* or species). In this way, the OA might offer useful resources for bringing such Hegelian insights – which I have attempted to reconstruct in this paper – to bear on contemporary discussions on norms and nature.

## Footnotes

1 A common way of formulating the issue is in terms of the distinction made by Wilfrid Sellars between two *logical spaces*: a normative space of reasons, and a non-normative realm of laws characterized by natural scientific explanation – with the latter often equated with “nature”. In Sellars’s words, “The naturalistic ‘thesis’ that the world can, ‘in principle,’ be described without using the term ‘ought’ or any other prescriptive expression, is a logical point about what is to count as a description *in principle* of the world” (Sellars 1957, § 79). For an overview, cf. De Caro & Macarthur (2010).

2 My attempt will move in the same direction as Cooper (2020) and Maraguat (2020). My focus, however, will be more on the kind of normativity involved in the notion of organisation, which I claim is foundational for Hegel. I will show how the notion of “organisation” is crucial to Hegel’s account of animal normativity and plays a more fundamental role than even the notion of “*Gattung*” or “species”. I consider my account compatible with recent analysis of higher forms of normativity in animal organisms, such as in the views of Ikäheimo (2021). I will return to this point later.

3 Before starting, one last introductory remark about my analysis. Current scholarship on Hegel and normativity displays two main interpretative lines. On the one hand, as stated above, many authors stress that Hegel’s notion of *geistig* or *spiritual* refers to a domain of normative intelligibility that falls *outside* the descriptive scope of the natural sciences and consequently, in an important sense, outside of nature (Brandom 2005, 2006, 2007, 2009, 2018; Pippin 2008, 2005; Pinkard 1994, 2012). In general, these accounts hold that normative properties cannot be said to be natural in any relevant sense – to use a phrase from Sellars, they “are not inferior, just different” (Sellars 1957, §§ 79). For these scholars, attainment of a position in which we assess things normatively is a social achievement, which involves “leaving nature behind” (Pippin 2002). One of the problems of this view is that it overlooks Hegel’s idea that some natural phenomena – especially those tied to living beings – constitutively involve some specific forms of normativity. On the other hand, however, interpretations of Hegel that investigate the idea of natural norms do so from a particular perspective, namely that of Neo-Aristotelian naturalism (Foot 2001, Hursthouse 1999, and especially Thompson 2008, 2013). This second line of interpretation is guided by a focus on the commonsense conception of life and organism and scrutinizes our “ordinary, natural or pre-scientific thought of things as alive” (Thompson 2008, 199). It thus performs “a kind of exposition of certain aspects of the ‘manifest image’” (Ibid., 10). This assumption is also problematic and in contrast with Hegel, since he does not seem to be singularly interested in the commonsense conceptual framework of our thoughts about living organisms. On the contrary, he seems to pursue a different project seeking a proper understanding of the *natural scientific concepts* of life. This explains the considerable effort Hegel exerted to stay up-to-date on contemporaneous research in the life sciences, such as physiology and comparative anatomy, as well as his attempts to incorporate the categories used in such studies into his work (Ferrini 2009, Breidbach & Engelhardt 2002). This interest on the part of Hegel is what motivates my own interest in the theoretical scaffolding of some current natural science theories of the organism. It seems faithful to the spirit of Hegel’s text to recover some of his insights by referring to current scientific categories, in this case coming from biological theory.

4 The most important works on “organisational accounts” are Moreno & Mossio (2015), Mossio et al. (2009), Garson (2017), Montévil & Mossio (2015), Bich & Mossio (2011), Mossio & Bich (2017), Mossio et al. (2016), Ruiz & Mirazo & Moreno (2004), Bich et al. (2016), Ruiz Mirazo et al. (2017). The OA is inspired by the idea of “autopoiesis” previously put forth by Maturana & Varela (1980) and aims to develop some underexplained notions of that earlier account while also solving some of its basic conceptual problems. I will leave aside the question of the compatibility of organisational and evolutionary accounts.

5 The distinction is inspired especially by the conceptual distinction between “material causes” and “efficient causes” drawn by Rosen (1991).

6 Montévil & Mossio (2015) gives a more formal account of the notion which specifies the role of “constraint” in terms of symmetries, while processes instead get specified in terms of thermodynamic flow. I will not dwell on this here, since the description outlined above is sufficient to bring into view the claims regarding normativity associated with this model.

7 For a closer description of the other constraints involved, cf. Montévil & Mossio (2015, 184): “Let us consider the production of an enzyme. As discussed above, an enzyme acts as a constraint on the reaction it catalyses. In turn, enzymes are themselves produced by and within the cell, through the translation process: ribosomes build the primary sequence of the future protein on the basis of the messenger RNA (mRNA) sequence, without consuming it. Since the ribosomes and the mRNA play a causal role while being conserved during this process, they both act as constraints (at a specific time scale) on the production of the enzyme. Consequently, the relationship between the enzyme, the ribosomes and the mRNA can be pertinently described as a dependence between constraints (in which the enzyme depends on both ribosomes and mRNA), insofar as all these entities satisfy the definition of constraint at specific time scales, which are considered jointly.”

8 Moreno & Mossio (2015), xxix. Montévil & Mossio (2015, 181) define “organisational closure in terms of the mutual dependence which exists among a set of entities that fulfill the role of constraints within a system”.

9 The distinction between “processes” and “constraints” is the main self-proclaimed innovation of the OA, which is understood to improve upon previous accounts of biological autonomy. This differentiates it from other frameworks, which understand “cohesion” as the key notion for the identification of life (Christensen & Bickhard 2002).

10 The model has also been extended to supra-organismal systems, such as symbiotic systems and ecosystems.

11 The standard narrative that appears in most writing by proponents of OA presents a lineage of organisational thinkers that usually starts with Kant and includes Claude Bernard, some figures in the cybernetics tradition, and Jean Piaget (the first to explicitly formulate the notion of closure), and then continues through the work of Jonas, Varela’s and Maturana’s insights on biological autonomy, and the theories developed by Robert Rosen and Stuart Kaufmann, who are considered the main representatives of the OA. Cf. Mossio & Bich (2017), Bich & Mossio (2011), Montévil & Mossio (2015). Michelini et al. (2018) expand this narrative.

12 For the history of the notion of “organisation” and reconstructions of how “organism” emerged as a central notion in modern biological accounts of life, cf. Cheung (2006, 2008, 2010) and Duchesneau (2018), Toepfer & Michelini (2016), Toepfer 2011, II 757 ff.

13 In his early Jena fragments on life, Hegel already defines the basic ontology of the organism as processual. He discusses the living individual as constituted by an “absolute organic process, the process of the individual” (*absoluter organischer Proceß der Proceß des Individuums*) (GW 6, 206).

14 Here I do not take up geological organisms, which are only *metaphorically* organisms. For Hegel, they lack “organic existence” (PN § 337 Z). On organisational differentiation as a marker of the organism, cf. “All organic being differentiates itself within itself, and maintains the unity of multiplicity” (PN § 344).

15 “This organic unity and perfect organisation *is* the animal” (GW 6, 205).

16 Hegel considers the individuation of differentiated parts a matter not of epistemic observation so much as of ontological individuation, cf. “the differentiation embodied in those organs is not one that pertains just to external reflection; such organs are rather the vital point of animal individuality”, WDL, 12.218 (717).

17 Hegel says the organism “belongs, as universal concept to the logical treatment” (“gehört als allgemeiner Begriff in die logische Betrachtung“ EZ‚ 908).

18 “Sensibility, Irritability and reproduction. The first two are abstract moments, the last one is the infinite combination (*unendiches Zusammenfassen*) of both (GW 24,2 926)”.

19 The language of “conditions of existence” was used extensively by George Cuvier, one of Hegel’s inspirations, to define the specific form of organisation in animals.

20 Cf. also Moreno & Mossio (2015, 82): “The closure of biological organisation provides the relevant grounding in which the concept of function can be adequately naturalised. In particular, it generates the norms that the traits subject to closure must fulfill in order to be functional: as we claimed, the organisational approach identifies these norms as the conditions under which the whole organisation…, and consequently each of its constituents, can exist. Thus, functional traits are all those whose causal effects contribute to the maintenance of the whole organisation.”

21 For criticism of the essentialist understanding of the notion of *Gattung* in Hegel, cf. Maraguat (forthcoming).

22 Some of these classical problems related to the OA are also addressed by Maraguat (2020), to which I am indebted. My taxonomy builds upon and extends Maraguat’s review of the standard difficulties raised by the OA. As will become clear, however, my reconstruction of Hegel’s response is different from his.

23 On this notion and its historical derivation from Cuvier, cf. Ferrini (2009) and Scholz (2020).

24 For a criticism, cf. Artiga & Martínez (2016) and the reply by Mossio & Saborido (2016).

25 I will say something about this in a moment. A full analysis of the notion, however, is behind the scope of this paper.

26 “Not every functional trait contributes to all possible regimes of self-maintenance of a given class, which means that an individual system can sometimes compensate for the breakdown of a component by shifting to a different regime of self-maintenance, in which the defective trait is no longer required. In contrast, some functional traits are indispensable, in that they are required for all regimes of self-maintenance that a member of a class could possibly adopt”, Mossio et al. (2009), 829–830. Cf. also Saborido & Moreno (2015, 92): “not every breakdown of a specific regime of self-maintenance will inevitably lead to the collapse of the system, and some biological traits may make a contribution to the maintenance of a self-maintaining organisation, even when these effects do not fulfill the functional presuppositions of the rest of the components”.

27 “A malfunctional trait is a structure unable to display the range of functional processes that other functional traits of the system presuppose, and, as a result, the system acts within a range of viability that is narrower than the range of viability that the system’s organisation presupposes” (Saborido & Moreno 2015).

28 Saborido & Moreno (2015) postulate a sub-system that is responsible for the regulation of the others. The sub-system triggers shifts between various regimes. The presence of this “meta-functional” system (Ibid., 90) raises something like a homunculus question: what prescribes the normal range of activity of such a system? How can we determine how such a sub-system *ought* to work?

29 As he states in the *1819–1820 Lectures*, when a “particular function” (*Funktion*) works against the general maintenance of the organism, this is sickness (GW, 24,1, 177); cf. “A system goes outside too much in his activity, so is it sickness” (*Tritt den System so sehr in Tätigkeit für sich heraus so ist Krankheit da*” (GW 24,1 178).

30 A view one could build upon to construct a Hegelian account of other forms of normativity, such as practical one (highlighted by Ikäheimo 2021, 62ff).

## Abbreviations

EL: Hegel, G.W.F. *Encyclopedia of the Philosophical Sciences in Basic Outline. Part I: Science of Logic*, trans. K. Brinkmann and D. O. Dahlstrom (Cambridge: Cambridge University Press, 2010)/*Enzyklopädie der philosophischen Wissenschaften im Grundrisse (1830). Erster Teil: Die Wissenschaft der Logik. Mit den mündlichen Zusätzen*, eds. E. Moldenhauer and K. M. Michel (Frankfurt: Suhrkamp Verlag, 1970).
GW 6: Hegel, G.W.F., *Jenaer Systementwürfe* I, (Gesammelte Werke 6), Ed. a K. Düsing and H. Kimmerle, Meiner, Hamburg 1975.
GW 24.1: Hegel, G.W.F., *Vorlesungen über die Philosophie der Natur 1. Nachschriften zu den Kollegien der Jahre 1819/20, 1821/22 und 1823/24* (Gesammelte Werke 24.1), ed. W. Bonsiepen (Hamburg: Meiner, 2012).
GW 24.2: Hegel, G.W.F., *Vorlesungen über die Philosophie der Natur 2. Nachschriften zu den Kollegien der Jahre 1825/26 und 1828* (Gesammelte Werke 24.2), ed. N. Hebing (Hamburg: Meiner, 2014).
PN: Hegel, G.W.F., *The Philosophy of Nature* (3 vols.), ed. and trans. M. J. Petry (London: Allen and Unwin, 1970).
WDL: Hegel, G.W.F., *The Science of Logic*, trans. G. di Giovanni (Cambridge: Cambridge University Press, 2010)/*Wissenschaft der Logik, Hauptwerke in sechs Bänden, Bänden 3 & 4* (Hamburg: Meiner, 2015).
